# Impact of organizational health-oriented strategies on employees' job performance, perceived medical mistrust as a moderator: A COVID-19 perception-based view

**DOI:** 10.3389/fpubh.2022.946946

**Published:** 2022-08-11

**Authors:** Mao Ye, Yu Chen, Yang Liu, Xiaohuan Li

**Affiliations:** ^1^School of Literature and Journalism, Xihua University, Chengdu, China; ^2^Agricultural and Rural Bureau of Shizhong District, Leshan, China

**Keywords:** organizational health-oriented strategies, psychological wellbeing, employee trust, job performance, perceived medical mistrust

## Abstract

After experiencing the COVID-19 pandemic, employees' health and well-being become a priority for firms. Organizational health-oriented strategies assist them in coping with health-related crises. Based on the social exchange theory, the present study attempts to determine the role of organizational health-oriented strategies in promoting employees' job performance. This study hypothesized that the organizations' health-oriented strategies positively correlate with employees' job performance. This study also assessed the mediating role of employees' psychological wellbeing and trust and moderating role of perceived medical mistrust. For the empirical examination, data of the present study was gathered from the textile sector in China. This study analyzed data through partial least square structural equation modeling (PLS-SEM). For this purpose, Smart-PLS software was used. The outcomes revealed that organizational health-oriented strategies positively enhance the employees' psychological wellbeing, trust, and job performance. Moreover, the results revealed that employees' psychological wellbeing and trust positively mediate the proposed relationships. This study found that perceived medical mistrust moderates the relationship between employees' psychological wellbeing and job performance. However, the findings revealed that perceived medical mistrust does not moderate the relationship between employees' trust and job performance. In addition, the present study's findings provide insights to the firms about the importance of health-oriented strategies. Moreover, this study's findings also serve the literature by providing important theoretical and practical implications.

## Introduction

Employees are an essential part of a firm as they can play a considerable role in its success (1). Poulis and Wisker (2) acknowledged that firms could utilize the intellectual abilities of employees as s strategic tool to differentiate themselves in the market. However, it is also noticed that the COVID-19 pandemic affects employees' physical and psychological health (3). Moreover, employees' overall performance is also adversely influenced when they have health-related issues. A previous study identified that after experiencing the COVID-19 epidemic, organizational health strategies are gaining more importance for employees' wellbeing and work performance (4). Further, they stated that after experiencing the COVID-19 pandemic, organizations must develop quick strategies to deal with a turbulent environment. Organizational health promotion activities are one of the important aspects of corporate social responsibility (5). In addition, they shed further light and said that firms should plan employees' healthcare strategies on a priority basis because it is a positive indicator of employees' wellbeing and job effectiveness.

A previous study noticed that firms' effective health strategies positively influence employees' physical and psychological wellbeing (6). Therefore, organizational health-oriented strategies are considered key to employees' wellbeing. Organizations should consider health-oriented strategies to promote employees' wellbeing and job productivity (7). Further, they stated that employees' satisfaction and loyalty level positively influences when organizations give importance to their wellbeing through proper strategies and procedures. Employees spend most of their day in the workplace, so it matters a lot to them how they are treated at the workplace by the organization (8). It is noticed that employees' trust was built when employees perceived that their organizations cared about their wellbeing (9). Further, they acknowledged that the trustfulness of employees is a positive indicator for boosting their job productivity and the overall performance of firms. In addition, organizational trust constructively influences the attitude and behavior of employees and plays a key role in strengthening the employment relationship between two parties (10). Further, they stated that organizations could save themselves from the undesired situation of contract breaches when the employment relationship is based on the trust of both parties. A previous study also commented on the adverse effects of psychological contract breach and said that this is the worst situation for the firm when employees perceive that their organizations are not fulfilling their obligations (11). Therefore, organizations must develop trustable relationships with their workforce (12). In addition, they point out that employees' strong psychological and emotional bond with their organizations build their trust and boost their job performance.

The COVID-19 pandemic adversely affects employees' work productivity (13). Moreover, in the early days of the pandemic, employees have to face lockdown situations, and they have to follow work from home strategies of firms. Further, they stated that the COVID-19 pandemic upset almost everyone's daily routine. However, it is noticed that COVID-19 vaccination was a big relief in this turbulent pandemic (4). The literature also identified that people's hesitancy from COVID-19 vaccination was reported globally (14). Further, they argue that this is a serious concern for organizations to prepare their workforce for COVID-19 vaccination. However, the perceived medical mistrust could be a possible reason for this hesitance of employees (15). It is acknowledged that perceived medical mistrust of employees adversely impacts their work attitude and behavior (16). Further, they stated that when employees perceive medical mistrust about some medical treatment, it negatively impacts health care quality. A previous study points out that individuals' uncertainty about vaccination is a huge hurdle to the success of vaccination programs (17). Further, they said that one recent survey about COVID-19 vaccination shows that more than 30% of people show hesitation and concern about having the vaccination. Moreover, the resistance of individuals makes it difficult to achieve projected targets for successful vaccination programs.

The present study serves the literature in four ways. First, this study provides insight into the role of organizational health-oriented strategies in employees' job performance. Based on social exchange theory (18), this study assumes that employees' job performance increases when organizations build health-oriented strategies. The present study adopts three important health-oriented strategies (preventive care, healthcare support, and health insurance) from literature (5). Second, this study tries to determine the mediating role of psychological wellbeing and employee trust between organizational health-oriented strategies and employees' job performance, respectively. Third, with the perspective of COVID-19 vaccination, the present study assumes that perceived medical mistrust of employees moderates the relationship between psychological wellbeing and job performance, and between employee trust and job performance. According to the authors' knowledge, this is first study that provides empirical insight on perceived medical mistrust with perspective of COVID-19 vaccination. Fourth, the findings of this study also have some important theoretical and managerial implications as well.

The remainder of this article is structured as follows: first, the present study introduces the key constructs of the theoretical framework and reviews the literature for hypothesis development. Second, the methodology of this paper is presented, and the results are discussed. In the next section, the discussion about study findings was discussed. Finally, the current study is concluded with future research directions and study limitations.

## Literature review

### Organizational health-oriented strategies

In this turbulent environment, the health-oriented strategies of firms are considered a key source of sustainable competitive advantage (19). Further, they pointed out the importance of health-oriented strategies and said that healthy employees could contribute effectively to attaining organizational goals. Moreover, the health-oriented strategies of firms are a positive signal to employees that their organizations care about their wellbeing. However, it is acknowledged that after the COVID-19 pandemic, it becomes more important for firms to make strategies on a quick basis to cope with this stumble situation (4). In addition, they stated that firms should have to redouble their energies and efforts to make health-oriented strategies for the betterment of their workforce.

A prior study points out that after experiencing the pandemic of COVID-19, organizations need to reschedule their strategies and plans for smoothing their day-to-day work routine (7). Further, they acknowledged that effective internal communication of firms could play a key role in the fruitfulness of these strategies. Moreover, the efficient role of the manager is also a valuable factor in coping with crises and building employees' confidence and trust. During health crises, effective internal communication of managers could serve as emotional support for employees by whom they can overcome their emotions such as fear and anxiety. A previous study draws firms' attention to important points for recovering from the crisis of the COVID-19 pandemic (6). One of these important points is that organizations can adopt key strategies to recover from the turbulence of an epidemic. There are three important organizational health-related strategies (preventive care, healthcare support, and health insurance) that can significantly affect employee wellbeing, satisfaction, and loyalty (5).

#### Preventive care

Preventive care comprises measures to prevent disease (20). Moreover, preventive measures are known as proactive actions to deal with upcoming healthcare crises. Some important preventive care measures include screening tests, check-ups, first aid training, vaccination, and supporting cessation programs (21). These preventive healthcare strategies could assist organizations in coping with uncertain health-related challenges. These proactive measures also paved the way for employees' constructive perception that their organizations care about their wellbeing.

#### Healthcare support

Healthcare support is the financial backing of organizations to cope with some undesirable health conditions of employees (5). Funds for disease cure, supporting recovery, and regenerative holidays are some important examples of healthcare support. It is noticed that healthcare support is one of the valuable organizational strategies for improving employees' wellbeing (22). Moreover, when employees perceive that their organizations are giving them healthcare support, they work more enthusiastically to fulfill organizational goals.

#### Health insurance

Health insurance is the financial support of firms to accommodate their employees in hard times (5). In addition, financial accommodations in case of illness, accident, or death are some examples of health insurance. Health insurance is an important organizational strategy to assist employees in dealing with their hard times (23). Moreover, employees' loyalty and trust build when they perceive that their organizations are always there to help them deal with health-related crises.

### Psychological wellbeing

A prior study defines psychological wellbeing as the extent to which employees have a positive emotional state which exhibits their level of happiness and satisfaction (24). Further, they stated that psychological wellbeing is the degree of emotional health and happiness people demonstrate when they feel satisfied with overall life functioning. A previous study identified three important aspects that define psychological wellbeing in more detail (25). The first aspect could be an individual's subjective experience of psychological wellbeing. In other words, it is an individual's point of view about how they perceive the degree of psychological wellbeing. Moreover, the second aspect indicates the degree of existence of positive emotions in an individual's mind and the absence of negative emotions. In other words, when individuals have a high level of psychological wellbeing, simultaneously, they experience low negative emotions (9). The third aspect is the objective nature of wellbeing which indicates the individuals' quality of life indicators that define their level of psychological wellbeing, such as material resources and social attributes.

It is acknowledged that employees' psychological wellbeing assists them in coping with stressful life events and help them survive the hard times of their life (26). Moreover, psychological wellbeing positively influences employees' work attitudes and behavior (9). In addition, the psychological wellbeing of employees boosts their creative thinking ability and enhances their level of work engagement. Moreover, when employees have a psychological bond with organizations, they perform beyond the expectations in the workplace. A prior study also pointed out the importance of psychological wellbeing and said that it motivates employees to do their work more effectively and efficiently (25).

It is noticed that the COVID-19 pandemic harms not only the physical health of individuals but also their psychological wellbeing (27). In addition, employees feel cynical after experiencing the negative outcomes of the pandemic, and they feel insecure about their future. A previous study also argued about the negative consequences of the COVID-19 pandemic on the emotional health of employees and said that this pandemic adversely influences employees' mental wellbeing and work attitude (28). Further, they point out that organizations must make proactive-basis strategies to deal with undesired crises. Based on the above-discussed literature, the present study assumes that organizational health-oriented strategies are positive signals for firms to enhance employees' psychological wellbeing. When employees perceive that their organizations care about their health and support them on hard days, they feel a sense of engagement and boost their psychological wellbeing. For empirical investigation present study hypothesize that

***H1:*** *Organizational health-oriented strategies have a positive relationship with psychological well-being*.

### Employee trust

Literature defines the term “trust” as an individual's confidence in another's reliability and integrity (10). In the organizational context, a trust could be defined as the belief and confidence of employees that their organizations are fair to them. The positive belief of employees constructively influences their work attitude and behaviors and encourages them to maintain long-term relationships with their firms. A prior study points out that psychological contract breaches can harm the relationship between employer and employee, resulting in negative outcomes for organizations (11). Further, they acknowledged that psychological contract breach leads to adverse consequences by decreasing employees' trust and work productivity.

Employees' trust in their organizations has a valuable role in boosting their commitment, satisfaction, and engagement (29). Additionally, a high level of employees' trust in their organizations constructively increases their job satisfaction and job performance (12). In addition, “ability, benevolence, and integrity” are three important dimensions of trustworthiness, adopted from Rousseau's definitions of reciprocity basis expectations (29). At the organizational level, ability, benevolence, and integrity mean employees assess their organization's trustworthiness based on the firm's competencies. For example, employees evaluated how their organizations fulfill their responsibilities, how firms take care of their employees' wellbeing, and how organizations follow moral principles, e.g., fairness and honesty.

It is noticed that the trust level of employees decreased due to the uncertainties and unpredictable changes experienced during the COVID-19 pandemic (29). Further, they stated that organizations should focus on trust repair strategies for employees to cope with crises after the COVID-19 pandemic. A previous study also stated that how organizations react and deal during some crises is a considerable question for firms (7). Further, they acknowledged that after the COVID-19 pandemic, employees' trust reduction is one of the adverse consequences of this epidemic. Moreover, they informed that organizations should focus on internal communications effectiveness and proactive health-oriented strategies to deal with health crises. Based on the above literature, the present study assumes that organizational health-oriented strategies positively build employee trust. Based on the social exchange theory, employees' trust boosts when they perceive that their organizations care for their wellbeing. For empirical investigation, the present study hypothesized that:

***H2:*** *Organizational health-oriented strategies have a positive relationship with employee trust*

### Job performance

Performance could be defined as how individuals perform their duties effectively and efficiently (30). In addition, according to a prior study's projections, performance is a two-dimensional model, including task performance and relationship performance (31). Moreover, task performance indicates job specification behaviors or prescribed behaviors, while relationship performance refers to behaviors that may be spontaneous or that may not be specifically job-oriented. Further, it is stated that employees' job performance is crucial to a firm's effectiveness and success (30). Further, they acknowledged that goal achievement is one of the important factors of effective job performance, which can, in turn, play a valuable role in organizational success.

A previous study points out that “task performance, interpersonal promotion, and work dedication” are three important dimensions of job performance (31). Task performance refers to the work contribution of employees in accomplishing the organizational goals and targets. Interpersonal promotion indicates the social aspects of employees' performance, such as increasing colleagues' morale, establishing and improving relationships with peers, and encouraging colleagues to boost their performance. Work dedication highlighted the employees' active work behavior such as discipline, selfless dedication, and the organization's welfare rather than self-interest. A previous study identified employees' psychological wellbeing as an important antecedent of their effective job performance (25). Further, they acknowledged that employees feel a sense of engagement and strong bond with their organizations when they perceive that their firms care about their wellbeing. A prior study also advocated the importance of employees' psychological wellbeing for their job performance and said that employees' work productivity enhances when their organizations care about their psychological wellbeing (8). In addition, employees' trust level increases when they perceive a psychological and emotional bond with their organizations (9). Further, they informed that employees' engagement and work productivity boost when they perceive that their organizations care about their wellbeing.

Based on the above-discussed literature, the present study assumes that the psychological wellbeing of employees and their trust level positively influences their job performance. Based on the social exchange theory, employees' work engagement and productivity are positively influenced when organizations care about their wellbeing. The organizational health-oriented strategies can also develop employees' trust, improving their job performance. For empirical investigation, the present study hypotheses that:

***H3:*** *Psychological wellbeing has a positive association with job performance*
***H4:*** *Employee trust has a positive association with job performance*
***H5:*** *Psychological wellbeing mediates the relationship between organizational health-oriented strategies and job performance*
***H6:*** *Employee trust mediates the relationship between organizational health-oriented strategies and job performance*.

### Perceived medical mistrust

A previous study defines medical mistrust as the extent to which people perceive their medical treatment as not trustable or secure (32). Further, they stated that medical mistrust is negative health-related behavior with some undesirable consequences for individuals and organizations. In addition, perceived medical mistrust is a big hurdle in improving health-related issues and coping with some uncertain health crises. The perceived medical mistrust is a negative behavior that can influence individuals' physical and emotional health (33). Further, they said medical mistrust is negative behavior and should be reduced by planning preventive strategies and approaches.

**Figure 1:**
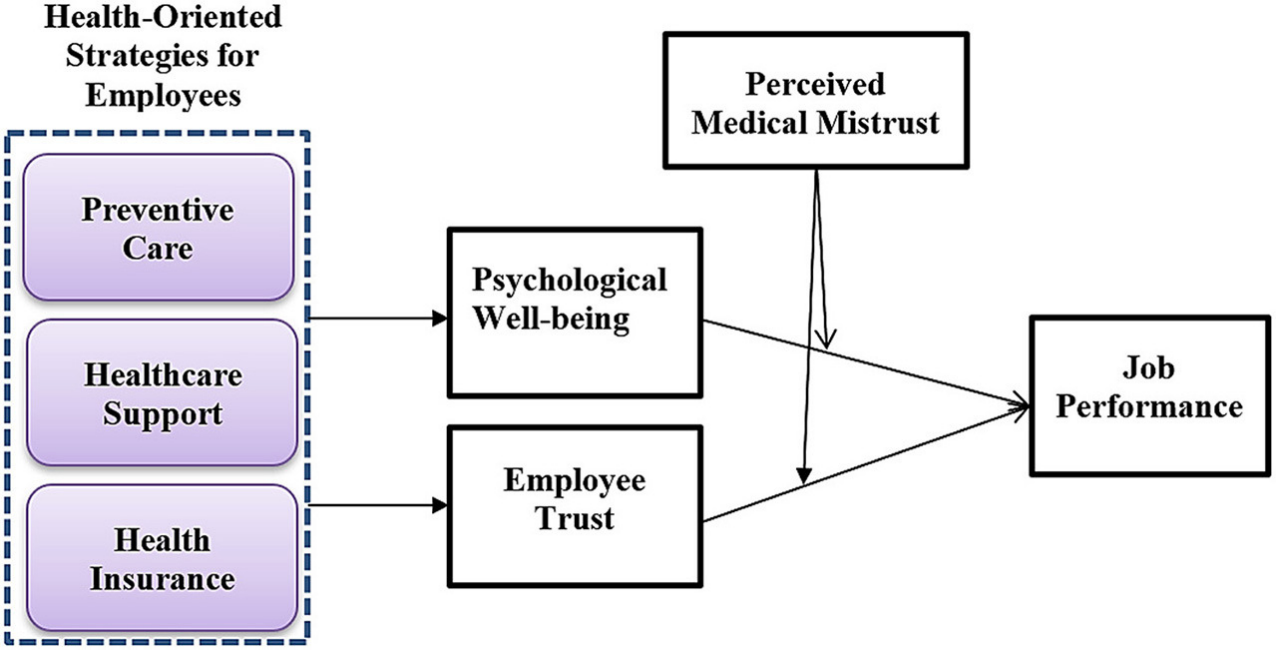
Conceptual framework.

The COVID-19 pandemic disturbs almost everyone's life routine and adversely affects individuals' physical and emotional health (15). Organizations have to make alternative strategies for working due to lockdowns and social distancing strategies. However, it is noticed that after the COVID-19 vaccination, organizations feel great relief to recover working conditions (4). After the COVID-19 vaccination was introduced, people's hesitation was reported globally (14). However, scholars noticed that perceived medical mistrust could be a possible reason for people's hesitancy toward COVID-19 vaccination (14, 15, 17). Based on the literature, the present study attempts to check the moderating role of perceived medical mistrust of employees. The present study attempts to check the role of perceived medical mistrust from the perspective of COVID-19 vaccination. The COVID-19 vaccination is a preventive measure to save employees from being victims of the pandemic. As in the above section, it is discussed that individuals feel hesitant and fear because they perceive that the vaccination might harm their health. This study tries to determine the employees' hesitant behavior toward COVID-19 under the medical mistrust construct and its impact on their job performance through interactional effects on their psychological wellbeing and trust. For instance, this study checks the moderating role of employees' perceived medical mistrust between their psychological wellbeing and job performance and their trust and job performance. Hence, the present study proposed the following hypotheses for empirical investigation, and Figure 1 represents this study's empirical model.

***H7:*** *Perceived medical mistrust moderates the relationship between psychological wellbeing and job performance*
***H8:*** *Perceived medical mistrust moderates the relationship between employee trust and job performance*

## Research methods

### Study design

This study followed a convenient sampling approach for the collection of data. In this regard, employees of different textile sectors in China were targeted. The author first approached the managers of the targeted textile sectors through phone calls and requested a face-to-face meeting at their convenient time. The firm managers who responded positively were shortlisted for further process. This way, the author fixed a meeting with the agreed managers. In the meeting, the author explained this study's whole objective. The author assured them about the data privacy, such as it would be used only for academic purposes, and promised to share the present study's practical implications with them at their request. Finally, the managers showed their consent, and the author distributed questionnaires personally among employees. The questionnaires were prepared with a cover letter. In this letter, the author explained the present study's objective and trusted them about their data confidentiality, such as accumulated results will be revealed instead of the individual level.

Moreover, the cover letter also confident the employees about the concept of right and wrong answers, such as their true answers will be treated right for this study instead of their consulted or copied answers; thus, consultation with their colleagues was not allowed during answering questionnaires. In this way, the employees filled out questionnaires with their true answers. The questionnaires were also translated into the Chinese language as understanding English was not an easy job for every employee. Hence, a team based-approach was incorporated for translations. In this regard, the author got the help of senior researchers and a Chinese language expert (34). As per the suggestion of the senior researchers, the author also filled out some translated questionnaires from the students to remove language difficulties for clear understanding. This way, the author finalized the questionnaires after being approved by senior researchers.

The author also decided to collect data in different turns to avoid common method bias. For this objective, a time lag data method was applied. The questionnaire also included a hidden code to identify the same respondents' in all turns. The author distributed the questionnaires in four turns. The questionnaires based on the independent variable (organizational health-oriented strategies) were distributed in the first turn. In this second turn, questionnaires were distributed based on mediator variables (psychological wellbeing and employee trust). In the third turn, the questionnaires regarding the dependent variable (job performance) were distributed, and in the fourth turn, the questionnaires regarding the moderator variable (perceived medical mistrust) were distributed among employees. In the first turn, the author distributed 1,500 questionnaires among employees and collected 1,110 questionnaires; after separating incomplete or non-useable questionnaires, the author got 10,34 complete and valid questionnaires in the first turn. In the second turn, after the 1-month gap, the author distributed 1,034 questionnaires by announcing that the employees who did not participate and did not properly fill questionnaires in the first turn to not participate in the second turn. The author got 750 questionnaires in the second turn, and by separating the incomplete or not useable questionnaires, the author finalized 599 valid and complete questionnaires in the second turn after recognizing the same respondents. In the third turn, the author distributed 599 questionnaires after a further 1-month gap. As per previous practice, the author made a similar announcement. The author got 487 questionnaires, and after separating non-useable questionnaires and verifying the same respondents, 439 questionnaires were finalized in this round. In the fourth turn, the author distributed 439 questionnaires after a further 1-month gap by adopting a similar practice based on the previous turn's collection; the author got 439 questionnaires. One questionnaire was found non-useable; hence it was discarded. In this way, the author finalized 438 valid and complete questionnaires. This way, the data collection procedure was completed in 4 months, started in November 2021 and finished in February 2022. Hence, this study is based on 438 sample sizes.

### Measures

The present study considered a Likert scale based on five points to measure participants' responses. In this scale, 1 denotes “strongly disagree,” 2 denotes “disagree,” 3 denotes “neutral,” 4 denotes “agree,” and 5 denotes “strongly agree.” The variables were measured based on prior study validated items. The independent variable organizational health-oriented strategies were measured with six items scale developed by Gorgenyi-Hegyes et al. (5). The mediated variables of psychological wellbeing were measured by three items scale adapted from the previous study Baker and Kim (8). The second mediator variable of employee trust was measured with four items adapted from Cook and Wall (35) and validated by Kelloway et al. (36). The dependent variable job performance was measured with four items scale developed by Walker (37) and validated by Chen and Silverthrone (38). The moderating variable of perceived medical mistrust was measured with a twelve-item scale adapted from Thompson et al. (39). The present study changed the group-based scale to an individual-based scale according to this study context. Appendix 1 (variable and items) represents this study's variables scale items.

## Results

### Assessment of measurement and structural model

The statistical outcomes of this study were analyzed through the partial least square structural equation modeling (PLS-SEM) method. PLS-SEM is taken because it is a variance-based method and differs from the covariance-based method (40). This study adopted PLS-SEM because it is appropriate for both studies, confirmatory and exploratory (41). Moreover, two techniques can be examined under structural equation modeling, covariance-based structural equation modeling (CB-SEM) and partial least square structural equation modeling (PLS-SEM). Both techniques have different roles, such as CB-SEM is appropriate for accepting or rejecting the theory, whereas PLS-SEM is appropriate for advancing or extending the theory (41). Moreover, PLS-SEM effectually handles small sample sizes. Hence, the data of this study was examined through PSL-SEM. For this purpose, Smart-PLS software was used. PLS-SEM measured data in two terms. The first term includes the measurement model, and the second term includes the structural path model.

The measurement model is based on two sections, i.e., reliability and validity. Reliability is assessed through Cronbach alpha, roh-A, composite reliability, and average variance extract (AVE) (41, 42). Table 1 explains model reliability. According to the criteria, the Cronbach alpha value should be more than 0.7 (43–48). All model variables such as independent variable (organizational health-oriented strategies), mediated variables (psychological wellbeing and employee trust), dependent variable (job performance), and moderated variable (perceived medical mistrust) Cronbach alpha values are 0.919, 0.838, 0.849, 0.838, and 0.933, respectively. These all values are according to the given standard of Cronbach alpha. Hence, Cronbach alpha values are accepted. Composite reliability values are accepted if these are above 0.7. Our study model all variables composite reliability values are 0.937, 0.902, 0.898, 0.893, and 0.942. These values are according to the given standard as all values are higher than 0.7. Hence, composite reliability values are also accepted. Similarly, the roh-A values of all variables are in the acceptable range and thus accepted. According to the criteria, the average variance extract (AVE) values should be above 0.5. Our models' variables have more than 0.5 AVE values, such as our models' variables independent variable (organizational health-oriented strategies), mediated variables (psychological wellbeing and employee trust), dependent variable (job performance), and moderated variable (perceived medical mistrust) AVE values are 0.712, 0.755, 0.689, 0.680, and 0.575, respectively. Hence, AVE reliability is achieved (49).

**Table 1:** Reliability and convergent validity of the study constructs.

**Construct**	**Item**	**Outer loadings**	**VIF**	**Alpha**	**roh-A**	**Composite reliability**	**AVE**
ET	ET1	0.800	1.789	0.849	0.850	0.898	0.689
	ET2	0.858	2.316				
	ET3	0.839	2.387				
	ET4	0.821	2.029				
HS	HS1	0.824	2.432	0.919	0.919	0.937	0.712
	HS2	0.862	2.963				
	HS3	0.874	3.093				
	HS4	0.846	2.636				
	HS5	0.864	2.866				
	HS6	0.792	2.042				
JP	JP1	0.654	1.309	0.838	0.860	0.893	0.680
	JP2	0.855	2.188				
	JP3	0.900	2.897				
	JP4	0.866	2.390				
PMM	PMM1	0.742	2.124	0.933	0.934	0.942	0.575
	PMM2	0.749	2.157				
	PMM3	0.723	2.081				
	PMM4	0.803	2.695				
	PMM5	0.761	2.406				
	PMM6	0.808	2.905				
	PMM7	0.757	2.202				
	PMM8	0.767	2.427				
	PMM9	0.724	1.972				
	PMM10	0.716	1.849				
	PMM11	0.775	2.541				
	PMM12	0.768	2.500				
WB	WB1	0.829	1.776	0.838	0.849	0.902	0.755
	WB2	0.883	2.098				
	WB3	0.894	2.131				

ET, Employee Trust; HS, Health-oriented Strategies; JP, Job Performance; PMM, Perceived Medical Mistrust; WB, Psychological wellbeing.

**Figure 2:**
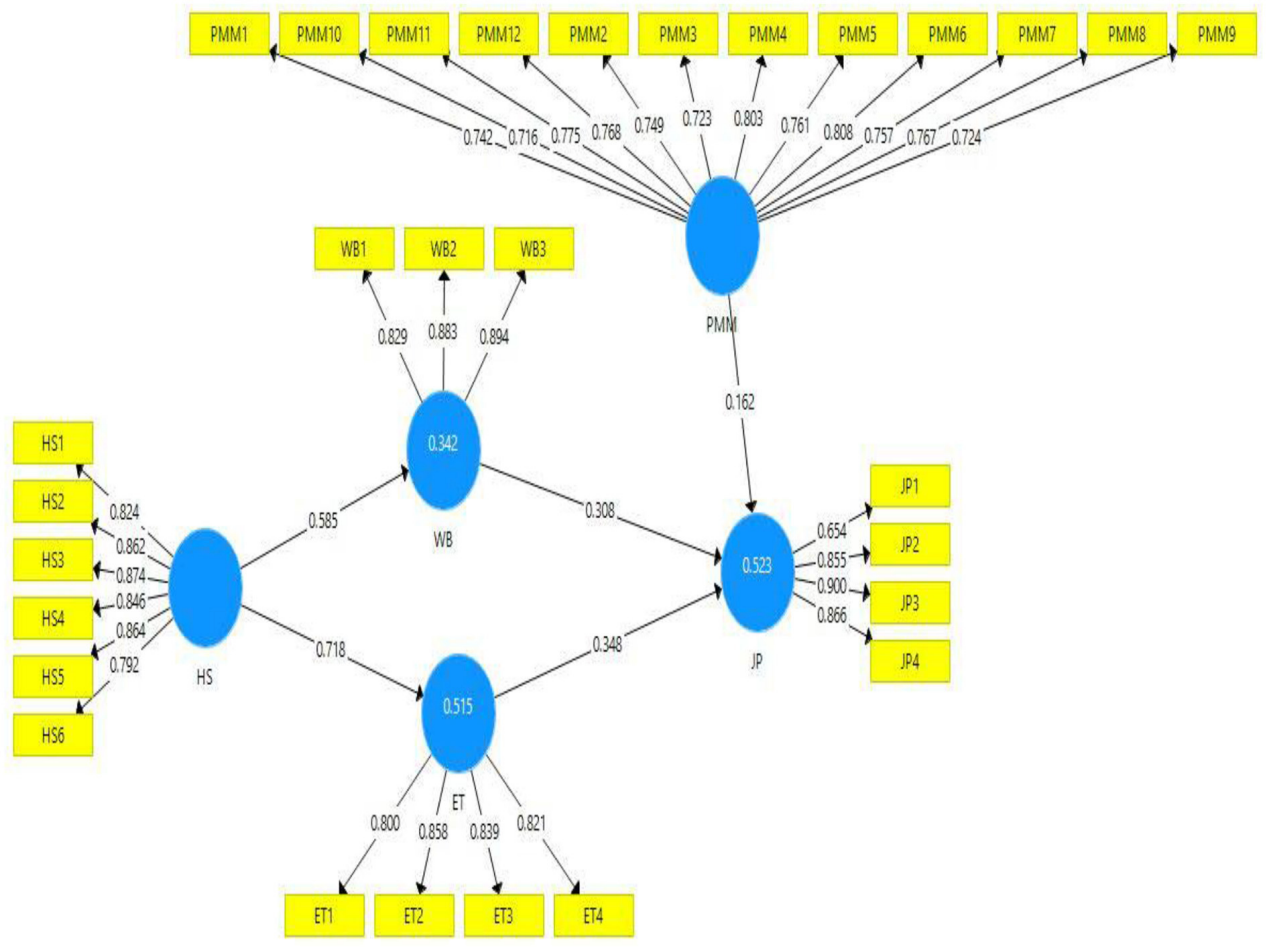
Path estimates.

The outer loadings of all variable items are also presented in Table 1. As per the criteria, the item's outer loading is accepted if >0.7 (41). The items of this study model variable have >0.7 outer loadings (Figure 2). Hence, it shows the strength of the models' reliability. Table 1 also presents variable item variance inflation factor (VIF) values. VIF explains the collinearity of the model constructs. A VIF value of <0.5 is considered a fit for the model (40). This study model variable “organizational health-oriented strategies” item HS3 has the highest VIF value (3.093). Hence, it confirmed that collinearity is not an issue in this study model.

The values of coefficient of determination (*R*^2^) were used to explain the strength of the study model (40). The level of variation explained in each endogenous construct, and the prediction accuracy of the study model are both shown by the *R*^2^ value of each endogenous construct. The latent constructs values near 0.5 and >0.5 explain moderate and substantial strength. This study's latent constructs (psychological wellbeing, employee trust, and job performance) *R*^2^ values are 0.342, 0.515, and 0.523, respectively. These values show that this model has moderate and substantial strength, a positive indicator of a good study model. Moreover, the latent variables values of Q^2^ are considered fit if greater than zero. The present study models latent constructs have Q^2^ values above zero. Hence, it confirms that the present has a significant model.

The present study examined the discriminant validity of the model by applying widely accepted approaches, for instance, Fornell-Larcker and heterotrait-monotrait (HTMT) ratio (42). The Fornell-Larcker values of the present study model constructs are presented in Table 2. It is examined by taking all constructs' square roots of AVE values (50). The Fornell-Larcker values are considered appropriate if all the above values are greater than their below values in the column. Table 2 explains that the above bold values are greater than the below values in the column. Hence, the outcomes are consistent with the Fornell-Larker criteria, confirming that discriminant validity is achieved. According to the threshold of HTMT, the HTMT value is considered appropriate if <0.85 (51, 52). Table 3 explains that this study model variable HTMT values according to the given threshold. Hence, HTMT discriminant validity is also confirmed.

**Table 2:** Discriminant validity (Fornell-Larker-1981 criteria).

**Construct**	**ET**	**HS**	**JP**	**PMM**	**WB**
ET	**0.830**				
HS	0.718	**0.844**			
JP	0.665	0.562	**0.824**		
PMM	0.744	0.635	0.607	**0.758**	
WB	0.637	0.585	0.628	0.606	**0.869**

ET, Employee Trust; HS, Health-oriented Strategies; JP, Job Performance, PMM, Perceived Medical Mistrust; WB, Psychological wellbeing.

Bold values shows the variable significance.

**Table 3:** Discriminant validity (HTMT).

**Construct**	**ET**	**HS**	**JP**	**PMM**	**WB**
ET	–	**–**	**–**	**–**	**–**
HS	0.812	–	**–**	**–**	**–**
JP	0.786	0.642	–	–	–
PMM	0.837	0.686	0.677	–	–
WB	0.752	0.662	0.734	0.677	–

ET, Employee Trust; HS, Health-oriented Strategies; JP, Job Performance, PMM, Perceived Medical Mistrust; WB, Psychological wellbeing.

This study's empirical analyses were conducted by applying 5,000 samples of the bootstrapping method. The direct, indirect, and total path outcomes are listed in Table 4 (42). The *t* and *p*-values are considered for hypotheses acceptance and rejection (41). The hypotheses results are listed in Table 5. According to proposition H1, organizational health-oriented strategies positively impact psychological wellbeing. The statistics results (*t* = 8.906, *p* = 0.000) revealed that organizational health-oriented strategies positively influence the employees' psychological wellbeing. Hence, H1 is accepted. The path value of H1 revealed that one unit change in organizational health-oriented strategies would result in a 0.585 change in employee psychological wellbeing. The H2 of this study proposed that organizational health-oriented strategies positively influence employee trust. According to the statistics outcomes (*t* = 14.330, *p* = 0.000), it is confirmed that organizational health-oriented strategies positively enhance employee trust. Thus, H2 is accepted. As per path value, one unit change in organizational health-oriented strategies would result in a 0.718 change in employee trust. The H3 of this study proposed that employee wellbeing has a positive relationship with their job performance and statistics results (*t* = 4.002, *p* = 0.000) confirmed that wellbeing have a positive impact on job performance. Thus, H3 is accepted. As per the H3 path value, one unit change in employee wellbeing would have 0.219 change in their job performance. The H4 proposed that employee trust positively impacts their job performance. The statistics results (*t* = 5.191, *p* = 0.000) revealed that employee trust positively enhances their job performance. Hence, H4 is accepted. The path value of H4 revealed that one unit change in employee trust would result in a 0.377 change in their job performance.

**Table 4:** Direct, indirect, and total path estimates.

**Direct path**	**Beta**	**SD**	***t***	**Confidence interval (95%)**	***f*^2^ effect size**	***p***
ET -> JP	0.377	0.073	5.191	(0.229–0.513)	0.096	0.000
HS -> ET	0.718	0.050	14.330	(0.605–0.803)	1.061	0.000
HS -> WB	0.585	0.066	8.906	(0.438–0.699)	0.521	0.000
PMM -> JP	0.122	0.068	1.789	(−0.008 to 0.257)	0.011	0.074
PMM*ET -> JP	0.061	0.043	1.426	(−0.032 to 0.139)	0.006	0.154
PMM*WB -> JP	−0.099	0.039	2.524	(−0.171 to −0.017)	0.020	0.012
WB -> JP	0.219	0.055	4.002	(0.110–0.324)	0.045	0.000
**Indirect path**	**Beta**	**SD**	***t***	**Confidence interval (95%)**		***p***
HS -> ET -> JP	0.271	0.058	4.702	(0.156–0.384)		0.000
HS -> WB -> JP	0.128	0.036	3.601	(0.063–0.203)		0.000
**Total path**	**Beta**	**SD**	***t***	**Confidence interval (95%)**		***p***
ET -> JP	0.377	0.073	5.191	(0.226–0.511)		0.000
HS -> ET	0.718	0.050	14.330	(0.611–0.807)		0.000
HS -> JP	0.399	0.064	6.200	(0.272–0.529)		0.000
HS -> WB	0.585	0.066	8.906	(0.447–0.702)		0.000
PMM -> JP	0.122	0.068	1.789	(0.000–0.264)		0.074
PMM*ET -> JP	0.061	0.043	1.426	(−0.035 to 0.137)		0.154
PMM*WB -> JP	−0.099	0.039	2.524	(−0.167 to −0.010)		0.012
WB -> JP	0.219	0.055	4.002	(0.113–0.328)		0.000

ET, Employee Trust; HS, Health-oriented Strategies; JP, Job Performance; PMM, Perceived Medical Mistrust; WB, Psychological wellbeing.

**Table 5:** Hypotheses testing.

**Hypotheses**		**Coefficient (beta)**	**S.D**	**t**	**Confidence interval (95%)**	***f*^2^ effect size**	***p***	**Status**
H1	HS -> WB	0.585	0.066	8.906			0.000	Supported
H2	HS -> ET	0.718	0.050	14.330	(0.605–0.803)	1.061	0.000	Supported
H3	WB -> JP	0.219	0.055	4.002	(0.110–0.324)	0.045	0.000	Supported
H4	ET -> JP	0.377	0.073	5.191	(0.229–0.513)	0.096	0.000	Supported
**Mediation** **hypotheses**		**Coefficient (beta)**	**S.D**	***t***	**Confidence** **interval (95%)**		***p***	**Status**
H5	HS -> WB -> JP	0.128	0.036	3.601	(0.063–0.203)		0.000	Supported
H6	HS -> ET -> JP	0.271	0.058	4.702	(0.156–0.384)		0.000	Supported
**Moderation** **hypotheses**		**Coefficient (beta)**	**S.D**	***t***	**Confidence interval (95%)**	*f*^2^ **effect size**	***p***	**Status**
H7	PMM*WB -> JP	−0.099	0.039	2.524	(−0.171 to −0.017)	0.020	0.012	Supported
H8	PMM*ET -> JP	0.061	0.043	1.426	(−0.032 to 0.139)	0.006	0.154	Not Supported

ET, Employee Trust; HS, Health-oriented Strategies; JP, Job Performance; PMM, Perceived Medical Mistrust; WB, Psychological wellbeing.

This study assessed the mediating role of psychological wellbeing and employee trust between organizational health-oriented strategies and job performance. For this purpose, H5 proposed that employee psychological wellbeing mediates the relationship between organizational health-oriented strategies and employee job performance. The statistics outcomes (*t* = 3.601, *p* = 0.000) revealed that psychological wellbeing mediates the relationship between health-oriented strategies and job performance. Moreover, the path value (0.128) of H5 confirmed that psychological wellbeing positively mediated between health-oriented strategies and job performance. Thus, H5 is accepted. The H6 proposed that employee trust mediates the relationship between health-oriented strategies and employee job performance and statistics outcomes (*t* = 4.702, *p* = 0.000), confirming the mediation role of employee trust in this relationship. According to the H6 path value (0.271), it is confirmed that employee trust positively mediated this relationship.

**Figure 3:**
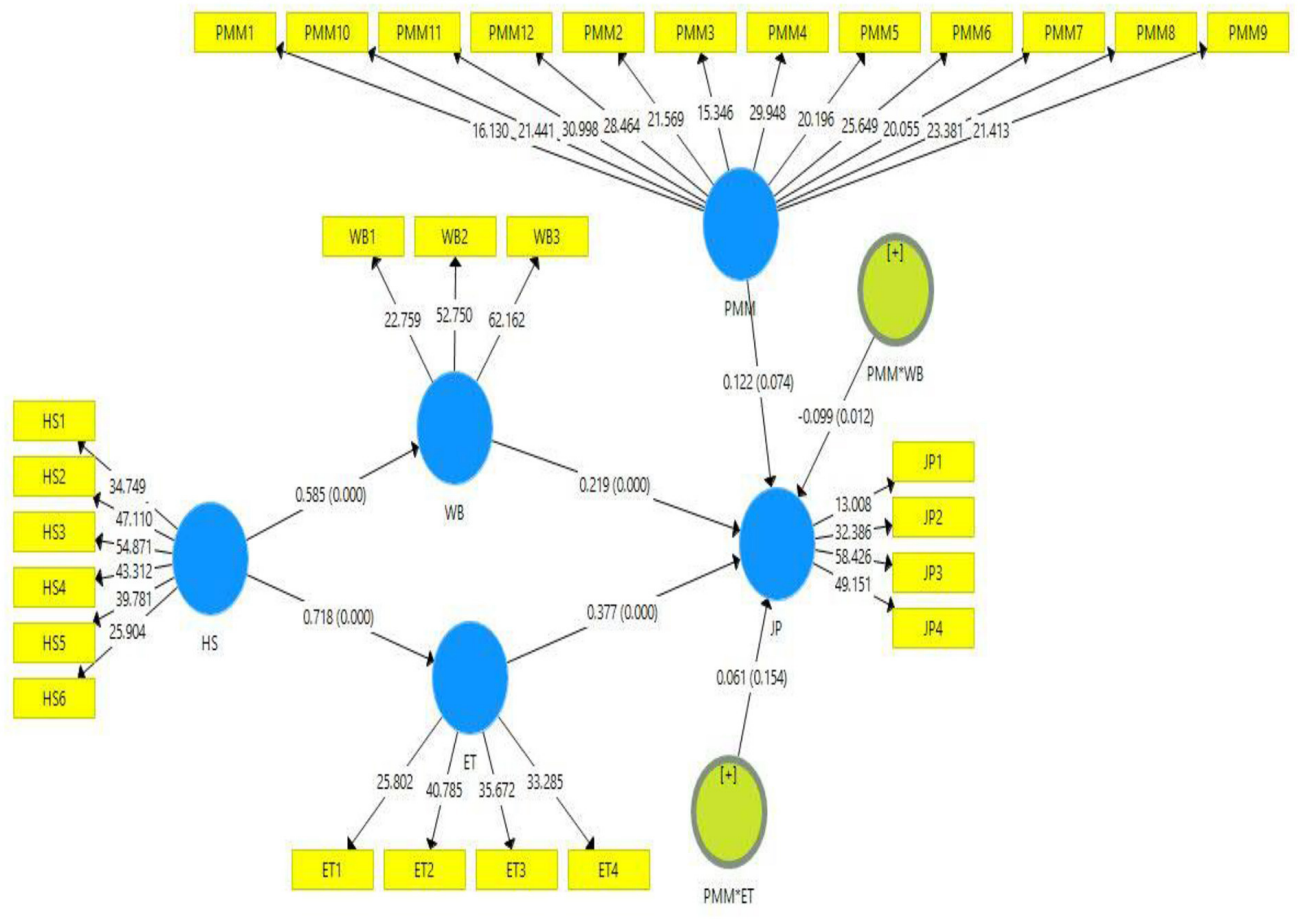
Bootstrapping estimates.

**Figure 4:**
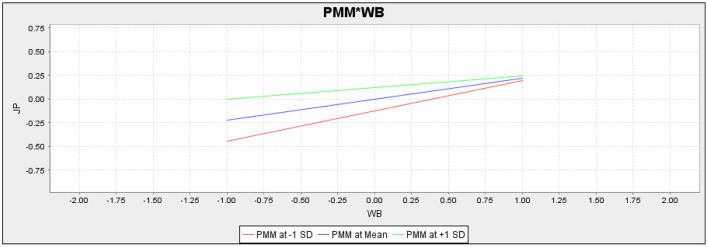
Moderation slope of PMM*WB.

**Figure 5:**
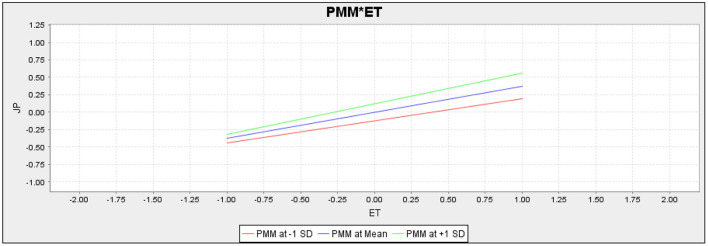
Moderation slope of PMM*ET.

Moreover, this study also assessed the moderating role of perceived medical mistrust in the relationship between psychological wellbeing and job performance and employee trust and job performance (Figure 3). The H7 proposed that perceived medical mistrust moderates the relationship between psychological wellbeing and job performance. According to the outcomes (*t* = 2.524, *p* = 0.012), it is confirmed that perceived medical mistrust moderates this relationship, and as per path value (−0.099), it is also confirmed that it negatively moderates this relationship. Hence, H7 is accepted. The H8 proposed that perceived medical mistrust moderates the relationship between employee trust and job performance. According to the statistics results (*t* = 1.426, *p* = 0.154), it is confirmed that perceived medical mistrust does not moderate the relationship between employee trust and job performance. Hence, H8 is rejected. Figures 4, 5 represent the moderation slope of H7 and H8.

## Discussion

Employees are considered valuable assets to the organization. Organizations can differentiate themselves in the market by utilizing employees' intellectual abilities. In this regard, this study explores the factors that enhance employees' job performance. This study develops a model based on social exchange theory and explores the role of organizational health-oriented strategies on employee job performance through mediating employees' psychological wellbeing and employee trust. This study investigated the direct association between organizational health-oriented strategies and employee psychological wellbeing and found that organizational health-oriented strategies positively influence the employees' psychological wellbeing. According to previous studies, when employees perceive that their organizations are caring about their health their satisfaction and engagement increase which in turn positively impacts their wellbeing (53, 54).

This study also explored the direct association between organizational health-oriented strategies and employee trust, and outcomes revealed that organizational health-oriented strategies positively enhance the employees' trust. The present study's findings confirmed that organizational health-oriented strategies positively increase employees' psychological wellbeing and trust. The direct association was also assessed between employee psychological wellbeing and job performance and employee trust and job performance. The outcomes revealed that employees' psychological wellbeing and employees' trust positively influence their job performance. Such as when employees have trust in management and have psychological wellbeing, they enthusiastically perform well in their job roles. The previous studies also noticed that the health-oriented strategies of firms are a positive signal for employees that their organizations care about their health and wellbeing (9, 12). Moreover, the employees feel confident about their firms, and their trust is also built positively, a positive signal for boosting their performance.

This study also found that employee psychological wellbeing and trust mediate the positive association between organizational health-oriented strategies and employees' job performance. This study shed light on the importance of organizational health-oriented strategies as if organizations have health-oriented strategies for their employees. Then in return, their employees have psychological wellbeing and trust in the organization that must enhance their job performance. This study also checked the moderating role of perceived medical mistrust of employees between employee psychological wellbeing and job performance and employee trust and job performance. The outcomes revealed that perceived medical mistrust negatively moderates the relationship between employee psychological wellbeing and job performance, such as high perceived medical mistrust weakening the relationship between employee psychological wellbeing and job performance. This study also found that perceived medical mistrust does not moderate the relationship between employee trust and employee job performance. The outcomes revealed that employees' psychological wellbeing is crucial, but organizations must reduce perceived medical mistrust as its interaction with psychological wellbeing reduces employee performance. Thus, organizations must facilitate their employees through health-oriented strategies and provide some training regarding the COVID vaccine's positive effects to reduce the negative perception of their medical mistrust.

## Theoretical and practical implications

This study has many theoretical and practical implications. Theoretically, this study extends the literature on organizational health-oriented strategies and job performance by taking the support of social exchange theory. Based on the social exchange theory, this study assumes that employees' job performance increases when organizations build health-oriented strategies. According to social exchange theory, the employees' performance enhances reciprocity when they perceive that their organizations care about their wellbeing. This positive perception of employees motivates them to take part in organizational activities more enthusiastically and do their best to enhance the productivity of the firm. The outcomes of this study confirmed that organizational health-oriented strategies positively influence the employees' psychological wellbeing and trust. The findings also authenticate that employees' psychological wellbeing and trust positively influence their job performance. This study also extended the literature on employee psychological wellbeing and employee trust as both play a mediating role between organizational health-oriented strategies and job performance. Moreover, this study serves the literature on employees' perceived medical mistrust, as it moderates the relationship between psychological wellbeing and employee job performance.

The current study offers several valuable implications for managers regarding practical contributions. This study provides guidelines to organizations and managers for advancing their health-related strategies to improve employees' work productivity and performance. First, organizations should realize the importance of the health and wellbeing of their employees because their health is very important for efficiently doing a job. Specifically, after experiencing the COVID-19 pandemic, the organizations have to take extra care of their workforce to cope with the turbulent consequence of the pandemic. When organizations develop health-oriented strategies to facilitate their employees, employees develop psychological wellbeing and trust in the organization, which positively increases their job performance. Second, the present study's findings also highlighted the importance of employees' psychological wellbeing and trust in increasing their performance. Third, this study points out that perceived medical mistrust could be a possible hurdle in the job performance of employees, as perceived medical mistrust of employees adversely impacts their work attitude and behavior. The workforce should also be provided proper psychological counseling by organizations to avoid potential consequences of perceived medical mistrust.

## Limitations

Like other social studies, this study also has limitations. First, the data of this study was gathered through a questionnaire survey method under a time lag approach to avoid common method bias and have a small data size. Hence, future research may conduct other methods for data collection, such as semi-structured questionnaires or interview methods. Additionally, future studies may adopt different techniques to avoid common method bias and enlarge the sample to strengthen and validate this study's outcomes. Second, this study checked the mediating role of employee psychological wellbeing and trust between organizational health-oriented strategies and job performance. Future research may check other mediators such as psychological ownership and emotional attachment to extend our study model. Third, this study examined the role of perceived medical mistrust as a moderator. Future research may check other moderators like employee cynicism and perceived organizational politics to validate the results of the present study. Moreover, this study observed data from textile sector employees in China. Future research should collect data from different sectors to verify the study outcomes. Future research may also conduct a similar study in western countries as results may vary due to cultural differences.

## Conclusion

Every organization is operating to make a profit. For this purpose, the organizations utilize all their resources to enhance organizational performance. Efficient human capital is a considerable asset of the organization that plays a crucial role in achieving organizational goals. After experiencing the COVID-19 pandemic, there is a need to prioritize employees' health and wellbeing. In this regard, this study developed a model under the support of social exchange theory to examine the role of organizational health-oriented strategies on employees' job performance through the mediation of employees' psychological wellbeing and employee trust. This study found that organizational health-oriented strategies positively influence employee psychological wellbeing and trust; in turn, employee psychological wellbeing and trust enhance job performance. Moreover, this study found that employee psychological wellbeing and trust positively mediate the relationship between organizational health-oriented strategies and employee job performance. This study also found that perceived medical mistrust negatively moderates the relationship between psychological wellbeing and job performance and does not moderate between employee trust and job performance. Hence, organizations should develop health-oriented strategies that facilitate the employees regarding their health issues. Employees' psychological wellbeing and trust in the organization would positively increase their job performance.

## Data availability statement

The original contributions presented in the study are included in the article/Supplementary material, further inquiries can be directed to the corresponding author/s.

## Author contributions

YL and XL: conceptualization. YC: data collection. MY: writing the draft. All authors read and agreed to the submitted version of the manuscript.

## Funding

This study was supported by the National Natural Science Foundation of China (General Program)-Research on the mechanism and mode of multi format linkage development of digital creative products (No. 71874142) and Sichuan Social Science Planning Project-Digital reconstruction and countermeasures of Wenchuan earthquake disaster memory based on public space (No. SC21B118).

## Conflict of interest

Author XL was employed by the organization (Agricultural and Rural Bureau of Shizhong District). The remaining authors declare that the research was conducted in the absence of any commercial or financial relationships that could be construed as a potential conflict of interest.

## Publisher's note

All claims expressed in this article are solely those of the authors and do not necessarily represent those of their affiliated organizations, or those of the publisher, the editors and the reviewers. Any product that may be evaluated in this article, or claim that may be made by its manufacturer, is not guaranteed or endorsed by the publisher.
