# Supplementary material for: Impact of organizational health-oriented strategies on employees' job performance, perceived medical mistrust as a moderator: A COVID-19 perception-based view
Source: Front Public Health. 2022 Aug 11;10:946946. doi: 10.3389/fpubh.2022.946946 (PMC9403412; doi:10.3389/fpubh.2022.946946)
Supplement: Supplementary file 1 [file Data_Sheet_1.docx]

**Appendix-1- Variable & Items**

***Health-oriented Strategies***

Preventive care

1. My organization provides screening tests and vaccination.

2. My organization provides first aid training.

Healthcare support

3. My organization provides health fund contributions.

4. My organization supports the recovery of health expenses.

Health Insurance

5. My organization provides financial support in case of illness.

6. My organization provides health insurance in case of accident or death.

***Psychological well-being.***

1. When dealing with these situations, I am satisfied with achieving self-fulfillment

2. When dealing with these situations, I am satisfied with achieving emotional health.

3. When dealing with these situations, I am satisfied with achieving personal goals and hopes.

***Employee trust***

1. Management at my firm is sincere in its attempts to meet the workers’ point of view.

2. Management can be trusted to make sensible decisions for the firm’s future.

3. Management at work seems to do an efficient job.

4. I feel quite confident that the firm will always try to treat me fairly.

***Job performance***

To what extent do you agree that

1. Quality of your performance is high.

2. Your productivity on the job is high.

3. The performance of your peers at their jobs is low compared with yourself doing the same kind of work.

4. Your performance at your job is high compared with your peers doing the same kind of work.

***Perceived medical mistrust (Revised)***

1. People have concerns about COVID-19 vaccination.

2. People do not trust doctors and vaccination staff.

3. People are suspicious of information from doctors and vaccination staff.

4. People do not confide in doctors and vaccination staff because it will be used against them.

5. Doctors and vaccination staff badly behave with people.

6. Doctors and vaccination staff do not take the medical complaints of people seriously.

7. I have personally been treated poorly or unfairly by doctors or vaccination staff because of my status.

8. People of lower status do not treat the same as higher status people by doctors and vaccination staff.

9. People of lower status do not receive the same medical care from doctors and vaccination staff as higher status people.

10. In most vaccination centers, people with different statuses receive different kinds of care.

11. Doctors and vaccination staff do not have the best interests of lower-class people in mind.

12. Doctors and vaccination staff sometimes hide information from patients who belong to the lower class.
